# From an electrophoretic mobility shift assay to isolated transcription factors: a fast genomic-proteomic approach

**DOI:** 10.1186/1471-2164-11-644

**Published:** 2010-11-18

**Authors:** Astrid R Mach-Aigner, Karin Grosstessner-Hain, Marcio J Poças-Fonseca, Karl Mechtler, Robert L Mach

**Affiliations:** 1 Gene Technology, Department of Gene Technology and Applied Biochemistry, Institute of Chemical Engineering, TU Wien, Getreidemarkt 9/166/5/2, A-1060 Wien, Austria; 2 Department of Genetics and Morphology, Institute of Biological Sciences, University of Brasilia, Campus Universitário Darcy Ribeiro, ICC SuL, AT-098, 70.910-900, Brasília-DF, Brazil; 3 Research Institute of Molecular Pathology, Vienna Biocenter, Dr. Bohrgasse 7, A-1030 Vienna, Austria

## Abstract

**Background:**

*Hypocrea jecorina *(anamorph *Trichoderma reesei*) is a filamentous ascomycete of industrial importance due to its hydrolases (e.g., xylanases and cellulases). The regulation of gene expression can influence the composition of the hydrolase cocktail, and thus, transcription factors are a major target of current research. Here, we design an approach for identifying a repressor of a xylanase-encoding gene.

**Results:**

We used streptavidin affinity chromatography to isolate the Xylanase promoter-binding protein 1 (Xpp1). The optimal conditions and templates for the chromatography step were chosen according to the results of an electrophoretic mobility shift assay performed under repressing conditions, which yielded a DNA-protein complex specific to the AGAA-box (the previously identified, tetranucleotide *cis*-acting element). After isolating AGAA-box binding proteins, the eluted proteins were identified with Nano-HPLC/tandem MS-coupled detection. We compared the identified peptides to sequences in the *H. jecorina *genome and predicted *in silico *the function and DNA-binding ability of the identified proteins. With the results from these analyses, we eliminated all but three candidate proteins. We verified the transcription of these candidates and tested their ability to specifically bind the AGAA-box. In the end, only one candidate protein remained. We generated this protein with *in vitro *translation and used an EMSA to demonstrate the existence of an AGAA-box-specific protein-DNA complex. We found that the expression of this gene is elevated under repressing conditions relative to de-repressing or inducing conditions.

**Conclusions:**

We identified a putative transcription factor that is potentially involved in repressing xylanase 2 expression. We also identified two additional potential regulatory proteins that bind to the *xyn2 *promoter. Thus, we succeeded in identifying novel, putative transcription factors for the regulation of xylanase expression in *H. jecorina*.

## Background

*Hypocrea jecorina *(anamorph *Trichoderma reesei*, [1]) is an abundant filamentous ascomycete. *H. jecorina *breaks down polysaccharides with a variety of hydrolytic enzymes that act synergistically [2,3]. Due to the high secretory capacity of this fungus (up to 100 g/L, [4]), *H. jecorina *has gained industrial importance and is employed both in the fermentative production of native extracellular enzymes and heterologous protein production. The hydrolases that are secreted by this fungus are applicable to many industries, including textiles (e.g., [5]), food and feed, (e.g., [6-8]), paper (e.g., [9,10]) and, most recently, biofuel production [11-13].

In 2006, we reported the identification of the main activator of hydrolases in *H. jecorina*, Xyr1 (Xylanase regulator 1) [14]. In addition to Xyr1, two transcription factors, Ace1 (Activator of cellulases 1) and Ace2 (Activator of cellulases 2), are potentially involved in the regulation of hydrolases in *H. jecorina *[15,16]. These two additional narrow domain transcription factors seem to directly modulate the mode of action of the general regulator Xyr1 [17,18]. The Carbon catabolite repressor Cre1 [19] has also been described as a wide domain repressor of particular hydrolase-encoding genes (e.g., [20,21]). Some years ago it was also postulated that another putative repressor protein exists that is specific for *xyn2 *(xylanase 2) [22]. An *in vivo *genomic footprinting approach identified the AGAA-box within the *xyn2 *promoter as a relevant *cis*-acting motif that is protected under glucose repressing conditions. Until recently, however, the corresponding *trans*-acting factor was not known.

Geyer and co-workers showed for the restriction endonuclease MboI that protein-DNA complexes could be identified with metal affinity chromatography, followed by mass spectrometry [23]. Later, affinity chromatography-SELEX (systematic evolution of ligands by exponential enrichment) was reported to be suitable for isolating transcription factors and modelling protein-DNA interactions [24,25]. In 2005, a streptavidin affinity assay was used to purify proteins of a known size that bind to the AP-1 (Activator protein-1) sequence (p47 and p49) in human cancer cells; these proteins were subsequently identified by sequencing the SDS-PAGE (sodium dodecylsulphate - polyacrylamide gel electrophoresis) protein bands [26].

Herein, we describe the isolation and identification of a transcription factor of unknown size with the combination of streptavidin affinity chromatography, a Nano-HPLC (high performance liquid chromatography)-tandem MS (mass spectrometry)-coupled detection system, and a genome based allocation. Our method is a fast genomic-proteomic approach for the identification of new and completely unknown transcription factors starting from a protein-DNA complex identified with an electrophoretic mobility shift assay (EMSA).

## Results

### The AGAA-motif within the *xyn2 *promoter is bound under glucose-repressing conditions

**Figure 1:**
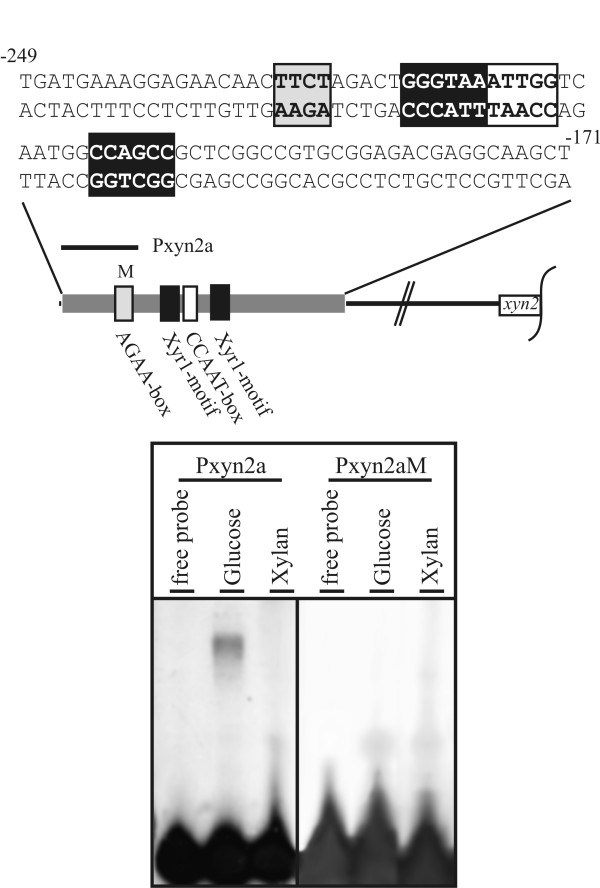
**Binding of potential regulatory proteins of *H. jecorina *to the AGAA-box within the *xyn2 *promoter**. First, 100 μg cell-free extracts derived from replacement experiments on glucose and xylan were subjected to an EMSA. Then, 10 ng of the radioactive-labelled oligonucleotides Pxyn2a (covering the area in the *xyn2 *promoter harbouring the AGAA-box (grey box; antisense strand), see Table 1) and Pxyn2aM (bearing a mutation of AGAA to CTCC (M), see Table 1) were used. Free probe indicates the sample lacking protein. Xyr1 indicates the Xyr1-binding sites (black boxes; GGGTAA on the sense strand and GGCTGG on the antisense strand). CCAAT indicates the binding site of the Hap2/3/5 complex (white box: antisense strand).

*In vivo *genomic footprinting data indicate that the transcriptional regulation of *xyn2 *expression involves the following *cis*-acting elements: a CCAAT-box, two Xyr1-binding motifs (GGGTAA and GGCTGG) known to bind Xyr1 (Xylanase regulator 1, [14]), and an AGAA-box [18,22]. Figure 1 summarizes the architecture of the *xyn2 *promoter. The AGAA-box, as well as the CCAAT-box and one Xyr1-binding motif, are located on the antisense strand. The AGAA tetranucleotide is fully protected under non-inducing conditions [22]. Cell-free extract protein from *H. jecorina *transferred to repressing (glucose) or inducing (xylan) conditions was subjected to an EMSA using radiolabeled probes. A protein-DNA complex was readily observed using cell-free extract obtained under repressing conditions and a short oligonucleotide containing the AGAA-box (Pxyn2a, see Table 1) (Figure 1). No protein-DNA interaction was detected when cell-free extracts from inducing conditions were used (Figure 1), supporting the idea that this part of the promoter is involved in repression. If the AGAA sequence is mutated CTCC (Pxyn2aM, see Table 1), no DNA-protein complex is formed under either repressing or inducing conditions (Figure 1). This result indicates that this part of the *xyn2 *promoter is essential for binding a transcription factor under repressing conditions.

**Table 1:** Oligonucleotides used in this study

Name	Sequence (5' - 3')	Employment
Bxyn2p250f	Biotin-TGATGAAAGGAGAACAACTTCTAGACTG	Affinity Chromatography
Bxyn2p250r	CAGTCTAGAAGTTGTTCTCCTTTCATCA	Affinity Chromatography
CKT067	CACTCCACATGTTAAAGGCGCATTCAACCAGCTTC	EMSA/Affinity Chromatography
CKT068	GAAGCTGGTTGAATGCGCCTTTAACATGTGGAGTG	EMSA/Affinity Chromatography
Exp2488F	GGATCCGACCGCATGGCGCACAAC	Construction of p2488DBD
Exp2488R	CTCGAGTCAACAGAATCCTCTCGGGTCG	Construction of p2488DBD
Exp3151F	GGATCCGAAGAAACCGCCAAGGCGC	Construction of p3151DBD
Exp3151R	CTCGAGAGATGTGTACGTCGGGTTTTC	Construction of p3151DBD
Exp7236F	GGATCCACACACGACCCCAACGCC	Construction of p7236DBD
Exp7236R	CTCGAGCGCGAGGGGGTTTCCATTC	Construction of p7236DBD
fltn2488f	GGTACCATGGCACAAGCCCTCGACATTTCC	Construction of p2488
fltn2488r	GCGGCCGCTCAACAGAATCCTCTCGGGTCGAAG	Construction of p2488
LPxyn2f-FAM	FAM-TGATGAAAGGAGAACAACTTCTAGACTGGGTAAATTGGTCAATGGCCAGCCGCTC	FAM-labelled EMSA
LPxyn2r	GAGCGGCTGGCCATTGACCAATTTACCCAGTCTAGAAGTTGTTCTCCTTTCATCA	FAM-labelled EMSA
LPxyn2Mf-FAM	FAM-TGATGAAAGGAGAACAACGGAGAGACTGGGTAAATTGGTCAATGGCCAGCCGCTC	FAM-labelled EMSA
LPxyn2Mr	GAGCGGCTGGCCATTGACCAATTTACCCAGTCTCTCCGTTGTTCTCCTTTCATCA	FAM-labelled EMSA
Pxyn2af	TGATGAAAGGAGAACAACTTCTAGACTG	Radioactive EMSA
Pxyn2ar	TGACCAGTCTAGAAGTTGTTCTCCTTTC	Radioactive EMSA
Pxyn2aMf	TGATGAAAGGAGAACAACGGAGAGACTG	Radioactive EMSA
Pxyn2aMr	TGACCAGTCTCTCCGTTGTTCTCCTTTC	Radioactive EMSA
transkr2488f	AGCTTCCACAAACATGACGCCG	Transcript analysis
transkr2488r	CATGGCGATTTCGAGCAGTCG	Transcript analysis
transkr3500f	CTCTTCAGGTCCTTATGAAGGTCG	Transcript analysis
transkr3500r	GAGTAGCTGTCCGATCCACG	Transcript analysis
transkr3151f	GATGTCTGAGGAATCTTCAAGCGC	Transcript analysis
transkr3151r	GGAGTCTTGCTTCGATTGCGG	Transcript analysis
transkr7236f	GTGTACCTGGACCTTGCGC	Transcript analysis
transkr7236r	CTGCTTCTCCTGGGGCG	Transcript analysis

The employment of the oligonucleotides used in this study is given. Underlined bases represent introduced restriction enzyme sites or bases added for labelling. Double underlined bases indicate introduced point mutations.

### Streptavidin-based isolation and identification of AGAA-binding proteins

**Figure 2:**
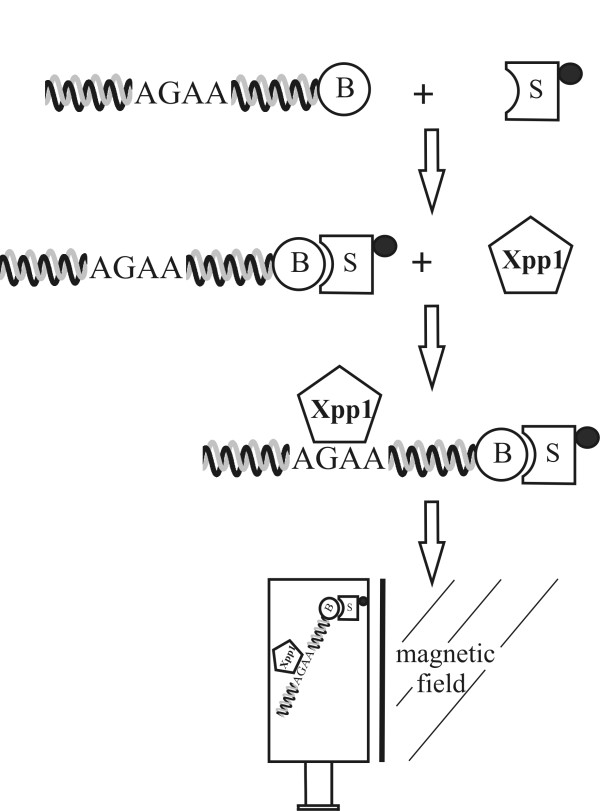
**Schematic drawing of the affinity chromatography assay**. The biotinylated (B) oligonucleotides bearing the AGAA-box were annealed and incubated with streptavidin particles tagged with a paramagnetic particle (S). Cell-free extracts from cells grown in glucose, containing the potential Xylanase promoter-binding protein (Xpp1), were added and incubated under the same conditions as those used for the EMSA (see Fig. 1). The Xpp1-DNA streptavidin complex was separated in a magnetic field according to the manufacturer's instructions (Miltenyi Biotec GmbH, Bergisch Gladbach, Germany).

The DNA-protein complex observed in the EMSA includes a putative repressor of *xyn2 *transcription (termed "Xpp1", Xylanase promoter-binding protein 1). To isolate this complex, the same batch of cell-free extracts from *H. jecorina *transferred to glucose was subjected to affinity chromatography. A schematic of the procedure is shown in Figure 2. Biotinylated versions of the same oligonucleotides used for the EMSA (Bxyn2p250, see Table 1) were incubated on ice with streptavidin beads (Figure 2). Under the EMSA reaction conditions, this mixture was incubated with the cell-free extract, which should include Xpp1 (Figure 2). After magnetic separation, the whole eluate was subjected to Nano-HPLC separation. The eluted peptides were monitored on the HPLC at a wavelength of 214 nm and directly applied to the mass spectrometer.

The tandem mass spectra were analyzed *in silico *as described above. 81 eluted proteins (see additional file 1) were identified and analyzed further with FGENESH V1 and GENEWISE 1. Three proteins with DNA-binding domains were identified and represent potential transcription factors (Table 2). These proteins (scan number 2488 (protein ID 122879), 3151 (protein ID 21557), 7236 (protein ID 108909)) were investigated in more detail and are hereafter referred to as "2488prp" (prp, promotor-binding protein), "3151prp", and "7236prp". The mass spectra for these three selected candidate genes are available online at: http://cores.imba.oeaw.ac.at/index.php?id=3731.

**Table 2:** Isolated and identified *H. jecorina *potential DNA-binding proteins of interest and verification of their transcript formation under repressing conditions in strain QM9414

Protein ID	Predictions according to FGENESH	Transcription
2488	Putative DNA-binding protein, helix-loop-helix	+
3151	DNA-binding domain, Zn-finger; BRAHMA-complex	+
7236	DNA-binding "high mobility group" protein	+

To verify that the genes encoding 2488prp, 3151prp, and 7236prp correspond to transcripts in *H. jecorina*, we performed RT-PCR using cDNAs obtained under inducing and non-inducing conditions. Using the primers listed in Table 1, we could clearly detect transcripts for the genes *2488prp*, *3151prp*, and *7236prp *(Table 2).

### Cloning, expression, and binding of putative AGAA-binding transcription factors of *H. jecorina*

**Figure 3:**
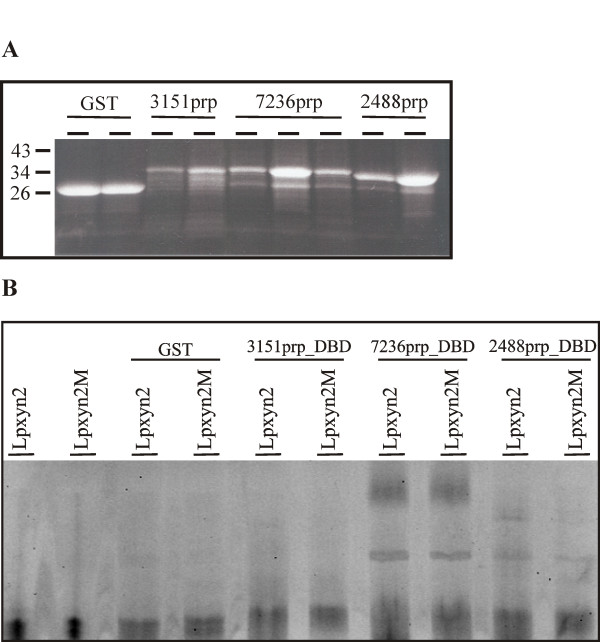
**Expression and binding of DNA-binding domains (DBD) of putative repressor proteins to the AGAA-box within *xyn2 *promoter of *H. Jecorina***. (A) SDS-PAGE of two clones of the DBD of 3151, three clones of the DBD of 7236, and two clones of the DBD of 2488. All clones heterologously expressed the GST-fusion proteins (3151prp, 7236prp, 2488prp). GST, expression of the GST-protein alone, as a control. A prestained protein ladder (Fermentas) was used. (B) EMSA with 100 ng of the DBDs expressed as GST-fusion proteins (see Fig. 3A). Finally, 15 ng of labelled oligonucleotides, one covering the respective area of the *xyn2 *promoter (Lpxyn2, see Table 1) and the other bearing a mutation in the AGAA-box (from AGAA to CTCC, Lpxyn2 M, see Table 1), were assayed alone (lanes 1, 2), with GST alone (negative control, GST), or with the thrombin-cleaved DBDs (3151prp_DBD, 7236prp_DBD, 2488prp_DBD).

Since transcripts for all three genes encoding the putative regulatory proteins were detected, the respective DNA-binding domains (DBD) were cloned and expressed as GST-fusion proteins using the pGEX-4T-2 vector and *E. coli *BL21-Gold. Production of the recombinant proteins was verified by SDS-PAGE (Figure 3A). All clones, two for the expression of the 3151prp DBD, three for the expression of the 7236prp DBD, and two for the expression of the 2488prp DBD, produced proteins of expected sizes (34 kD for 3151prp, 36 kD for 7236prp, and 32 kD for 2488prp). Two clones expressing GST alone were applied as controls and also produced bands of the correct size (26 kD).

The thrombin-cleaved DBD constructs were analyzed with an EMSA using an oligonucleotide covering the whole *xyn2 *promoter (Lpxyn2, see Table 1) or an oligonucleotide bearing the AGAA-box mutated to CTCC (Lpxyn2 M, see Table 1). FAM-labelled probes seem to demand longer probes: a FAM-labelled (shorter) Pxyn2a only gave a weak shift (data not shown). No shift was observed with the GST control or the 3151prp DBD (Figure 3B). The 7236prp DBD yielded two protein-DNA complexes, but these are rather non-specific, as they also formed with the mutated probe (Figure 3B). In contrast, the 2488prp DBD produced one slow-migrating shift that was not observed with the mutated probe (Figure 3B), indicating the formation of an AGAA-box specific DNA-protein complex. This candidate was analyzed in more detail.

### The binding of 2488prp to the *xyn2 *promoter of *H. jecorina*

**Figure 4:**
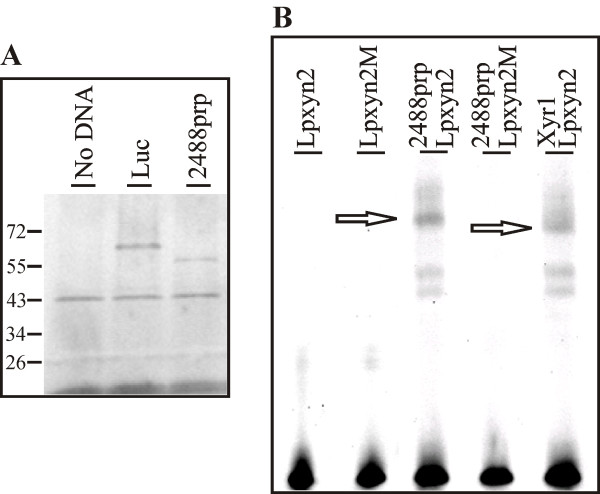
***In vitro *translation and binding of the 2488 putative repressor protein**. (A) SDS-PAGE of *in vitro *translated and FluoroTect™Green labelled proteins: a negative control reaction containing no DNA template (no DNA), a positive control reaction for the expression of luciferase (Luc), and the expression of 2488prp (pMPF2488 as DNA template). A prestained protein ladder (Fermentas) was applied. (B) EMSA using 60 ng of *in vitro *translated, unlabelled 2488prp (see Fig. 4A). Then, 15 ng labelled oligonucleotides, one covering the respective area of the *xyn2 *promoter (Lpxyn2, see Table 1), and another bearing a mutation from AGAA to CTCC (Lpxyn2 M, see Table 1) were applied without protein sample (lanes 1, 2). *In vitro *translated Xyr1 was used as a positive control.

Because 2488prp DBD produced an AGAA-box specific shift in the EMSA (Figure 3B), the corresponding gene was subjected to *in vitro *translation. The accumulation of product from the *in vitro *translation was confirmed with SDS-PAGE of the FluoroTect™Green-labelled proteins (Figure 4A). A negative control reaction containing no DNA template produced no specific protein, but a positive control reaction containing a plasmid that codes for luciferase gave a protein band of the correct size (61 kD) (Figure 4A). Using the plasmid pMPF2488 as template in an *in vitro *translation experiment was also found to yield a protein band of the expected size (54 kD) (Figure 4A). Background bands of 42 kD (rabbit reticulocyte lysate protein) and 18 - 25 kD (aminoacyl tRNAs) are associated with the *in vitro *translation procedure (see manufacturer's guidelines). We then performed an EMSA using the *in vitro *translated, unlabelled 2488prp and the labelled oligonucleotides (the same as for the EMSA with DBD). No DNA-protein complex formed with the mutated probe (Lpxyn2 M, see Table 1) (Figure 4B), but one specific shift was observed with the probe lacking mutations (Lpxyn2) (Figure 4B, indicated by an arrow). This shifted band migrated more slowly than the positive control shifted band using *in vitro *translated Xyr1 (Figure 4B, also indicated by an arrow). Xyr1, a general hydrolase activating transcription factor in *H. jecorina *[14], was recently reported to bind the *xyn2 *promoter [18], acting as a *cis*-acting element near the AGAA-box, which is also present on the applied oligonucleotide. The two shifts with the fastest mobility in the reactions containing oligonucleotide Lpxyn2 and *in vitro *translated 2488prp or Xyr1 (Figure 4B) are background. These shifts also appear if the *in vitro *translation mixture is given no DNA-template or the luciferase protein (data not shown). Therefore, we conclude that 2488prp binds the AGAA-box of the *xyn2 *promoter *in vitro*.

### Characterisation of *2488prp*

The putative *2488prp *gene encodes a protein of 505 amino acids with a predicted molecular mass of 55 kD. Sequence alignments indicate that the entire 2488prp protein has significant sequence similarity to hypothetical proteins in *Nectria haematococca *(GeneID: 9678588; 43%), *Gibberella zeae *(GeneID: 2791570; 43%), *Neurospora crassa *(GeneID: 3874038, 37%), *Podospora anserina *(GeneID: 6189947, 37%) *Chaetomium globosum *(GeneID: 4395593, 37%), *Magnaporthe grisea *(GeneID: 2675104, 36%). The most similarity was found in the C-terminal part of the protein (compare additional file 2), which includes a helix-loop-helix domain (HLH-superfamily; [27]) with an overall length of 67 amino acids (aa 397 to 461). A detailed domain analysis according to [28] revealed a basic DNA binding region (aa 397 to 406) N-terminal to two alpha-helices separated by a loop region (aa 409 to 461). The basic N-terminal region is thought to mediate high-affinity DNA-binding [29], whereas the helix-loop-helix region functions as a dimerization interface [30]. A glutamic acid at position 404 strongly indicates that 2488prp belongs to the group of E-box binding HLH proteins (3). The classical E-box is a hexameric palindrome. Interestingly, the AGAA-box overlaps with a hexameric palindrome (TCTAGA on the sense strand). Only the antisense part of this potential E-box has been reported to be protected according to *in vivo *footprinting experiments [18,22]. The sense strand cannot be analyzed because the sequence extension in the linker-mediated PCR is fully determined at the TATA-box (unpublished data, Würleitner E. and Mach R.L.).

### Transcription of *2488prp *during cultivation in different carbon sources

In *H. jecorina*, the expression of the hydrolytic enzyme-encoding genes, such as *xyn1*, *xyn2*, *bxl1*, *cbh1 *(cellobiohydrolase 1), *cbh2 *(cellobiohydrolase 2), *egl1 *(endoglucanase 1), or *bgl1 *(β-glucosidase 1), is regulated by the general activator Xyr1, regardless of the carbon source or inducing substance [14,31]. Nevertheless, different expression/induction patterns for these genes have been observed [17,22]. We examined, whether the transcription of this putative regulatory protein depends on the presence of certain induction signals or various carbon sources.

After pre-cultivation, the mycelium of *H. jeco*rina was transferred to medium lacking a carbon source (de-repressing conditions) or to media containing 1% (w/v) glucose, glycerol (repressing conditions), D-xylose or xylan or 1.5 mM sophorose or xylobiose (inducing conditions). Cultures were incubated for 3, 5, and 24 hours. After RNA-extraction followed by cDNA synthesis, the transcript levels were analyzed via real-time PCR.

**Figure 5:**
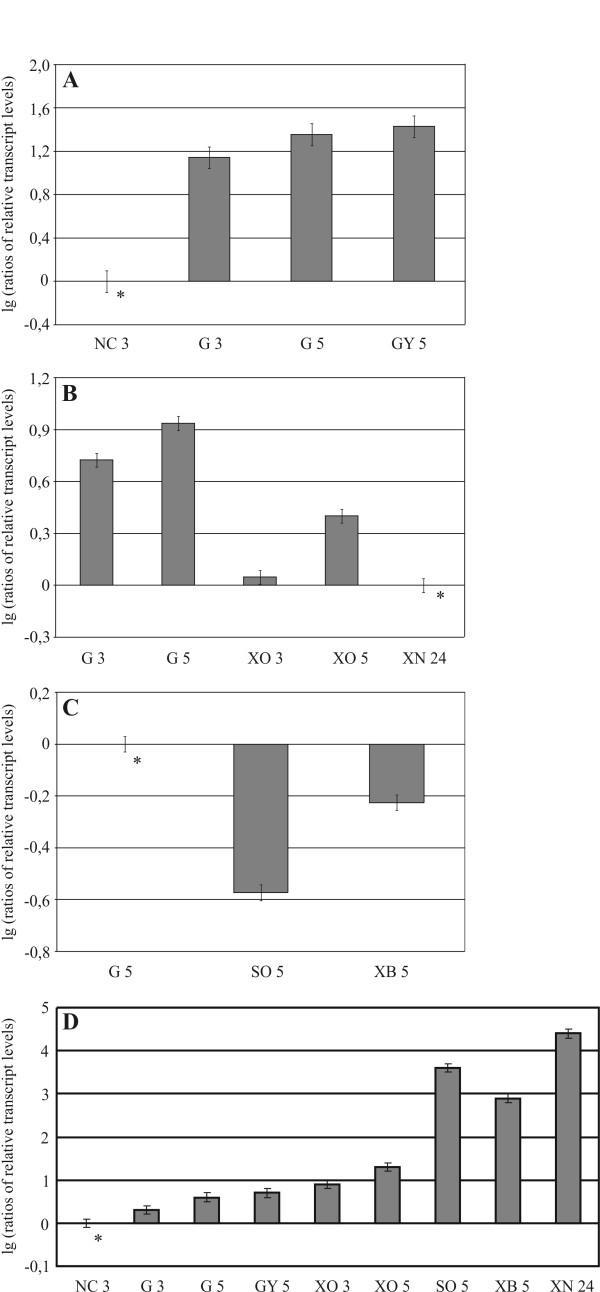
**Transcription analysis of carbon source-dependent regulation of *2488prp *and *xyn2 *transcription**. (A) Transcription of *2488prp *under repressing conditions. The *H. jecorina *strain QM9414 was pre-cultured on glycerol and then transferred to MA media lacking a carbon source (NC) or containing 1% (w/v) glucose (G) or glycerol (GY), and incubated for 3 and 5 hours, respectively. (B) Transcription of *2488prp *under growth conditions. Replacement of QM9414 was performed to 1% (w/v) glucose (G), xylose (XO) or xylan (XN) as sole carbon source and incubated 3, 5 and 24 hours, respectively. (C) Transcription of *2488prp *under inducing conditions. Replacement of QM9414 was performed to 1% (w/v) glucose (G), 1.5 mM sophorose (SO) or xylobiose (XB), and incubated 5 hours. (D) Transcription of *xyn2 *under various carbon source conditions. The rates of transcription for the above mentioned (growth) conditions are summarized. The relative transcript levels are given on decade logarithmic scale (lg). The data presented are means of results from three independent experiments. Error bars indicate the standard deviations. The asterisk indicates the reference sample.

We observed that the abundance of the *2488prp *transcript increases in the presence of carbon sources that repress hydrolase expression, such as glucose or glycerol, relative to de-repressing conditions (Fig 5A). Similar observations were made when comparing inducing conditions, such as growth on D-xylose or xylan or in media containing xylobiose or sophorose, with transcript forming in media containing glucose (Figure 5B, C). In all cases, it was clear that the transcription of the putative repressor *2488prp *gene was downregulated. These data strongly indicate that *2488prp *transcription is upregulated under repressive conditions and that this protein functions as a repressor of hydrolase transcription.

## Discussion

In the last few decades, several techniques have been developed to identify new DNA regulatory elements. Both *in vitro *methods, such as EMSA with cell-free or nuclear extracts ([32] and citations therein), and *in vivo *approaches, such as the *in vivo *genomic footprinting assay ([33,34] and citations therein) and promoter deletion analyses, have enabled the identification of numerous *cis*-acting elements.

The isolation and identification of the corresponding *trans*-acting factors is still challenging, however. Until recently, affinity chromatography based methods for the isolation of transcription regulators demanded large amounts of samples (e. g. cell-free extract proteins) and/or laborious pre-purifications steps (e. g. heparin-sepharose purification (e. g. [35,36])). Furthermore, additional purification steps, such as one- or two-D gel electrophoreses, were necessary to obtain at least semi-purified protein, which could thereafter be subjected to nanospray MS/MS fragmentation sequencing [26]. Although such methods led to the identification of transcription factors, they are labour-intensive and not suitable for high-throughput analyses.

Recently, the yeast one hybrid (Y1H) system was established, offering an *in vivo *method of screening respective transcription factors in a living cell [37]. This method depends on the completeness of the cDNA library, however. Thus, cDNAs of rare mRNAs, such as those of transcription factors and narrow domain regulators, are often missed. Furthermore, if the DNA binding depends on posttranslational modifications or the presence of additional interacting factors, it is even more likely that relevant interactions can be missed. The Y1 H system is also laborious and time-consuming (e. g. isolation of rare mRNAs, cDNA library constructions) and therefore only partially useful for high-throughput screening approaches. In addition, Y1 H and Y2 H approaches are known to be prone to false positives (e. g. [38,39]).

The method described in this study requires only small amounts of protein extract and the preceding extraction procedure is easy and fast. It combines a single-step micro affinity chromatography purification based on prior *in vivo *and/or *in vitro *protein-DNA-interaction data and high-resolution analyses. The Nano-HPLC/tandem MS-coupled detection allows the separation and identification of up to 500 proteins in one sample. Such a large number of identified candidate proteins enable the allocation of protein complexes interacting with the respective DNA target. We required that the putative transcription factor contain a predictable DNA binding domain to be considered for further analyses. Comparing protein extracts derived from different growth or environmental conditions and/or from a time-course experiment could provide information on the dynamics of protein networks interacting with the DNA. Finally, such a technique will permit mutational analysis of *cis*-acting DNA elements.

In this study, the most promising protein that was identified (2488prp) is an E-box binding HLH protein. The classical E-box is a hexameric palindrome. Interestingly, the AGAA-box overlaps with a hexameric palindrome (TCTAGA on the sense strand). Until this work, only the antisense part of this potential E-box was reported to be protected in footprinting experiments *in vivo *[18,22], because the sense strand cannot be analyzed due to sequence extension in the linker-mediated PCR is fully determined at the TATA-box (unpublished data, Würleitner E. and Mach R.L).

## Conclusions

The method developed here should be applicable to other high-throughput experiments, given available genome sequence databases. It permits the assignment of a promoter-binding protein (in this study, Xpp1) to its previously identified *cis*-acting element (the AGAA-box) under various conditions (glucose-repressing conditions) for the expression of a certain gene of interest (*xyn2*). The described genomic-proteomic approach facilitates the fast one-step isolation and identification of an unknown promoter-binding protein. In addition, this method assists in assigning the promoter-binding protein to a certain gene regulatory function.

## Methods

### Strains and growth conditions

The ascomycete *H. jecorina *(*T. reesei*) QM9414 (ATCC 26921; a cellulase hyper-producing mutant derived from the wild-type strain QM6a [40]) was used throughout this study and maintained on malt agar. For replacement experiments, mycelia were pre-cultured in 1-L-Erlenmeyer flasks on a rotary shaker (250 rpm) at 30°C for 18 h in 250 mL of Mandels-Andreotti (MA) medium [41] supplemented with 1% (w/v) glycerol as a sole carbon source. 10^8 ^conidia per litre (final concentration) were used as the inoculum. Pre-grown mycelia were washed and then equal amounts were resuspended in MA media containing 1% (w/v) glucose, glycerol, xylose or xylan as the sole carbon source or supplemented with 1.5 mM sophorose or xylobiose or without any carbon source.

*E. coli *JM109 (Promega, Wisconsin, US) was used for the propagation of plasmid vectors, and the strain BL21-Gold (Stratagene, La Jolla, CA) was used as the host for the production of GST (Glutathion S-transferase) fusion proteins.

### Electrophoretic mobility shift assay (EMSA)

Radioactive EMSA synthetic oligonucleotides (VBC, Vienna, Austria) (Table 1) were used. After annealing, double stranded oligonucleotides were end-labelled with (*-32P)-dCTP using Klenow Polymerase (Promega) and purified with non-denaturing PAGE (polyacrylamide gel electrophoresis). The binding assays and PAGE experiments were performed essentially as described in [41]. Binding was achieved by incubating 100 μg of cell-free extract protein with 5 ng of labelled fragment for 15 min on ice. Cell-free extracts were prepared as described previously [41].

For fluorescent EMSA, the synthetic FAM-labelled oligonucleotides (MWG Biotech, Ebersberg, Germany) were annealed with their complementary oligonucleotides (Table 1) by cooking them in 200 mM Tris/HCl (pH 7.5) for 5 min and then letting them cool slowly to room temperature. The binding assay and PAGE experiments were performed as described previously [41]. Binding was achieved by incubating 100 ng of the GST fusion proteins or 60 ng of *in vitro *translated, unlabelled 2488prp (putative repressor protein) with 15 ng of labelled fragment for 15 min on ice. *In vitro *translation of the complete 2488prp protein was performed using the TNT Quick Coupled Transcription/Translation System (Promega) according to the manufacturer's instructions starting from the plasmid pMPF2488, which inserts the *2488prp *structural gene into the vector pTNT (Promega). To verify the completion of the translation process by a SDS-PAGE, both 2488prp and luciferase (a control template DNA provided by Promega) were labelled using FluoroTect™Green (Promega). Gels (EMSA and SDS-PAGE) were analysed using a Typhoon 8600 variable mode imager (Amersham Bioscience, part of GE Healthcare, CT, US).

### RNA-extraction, reverse transcription, transcript analyses, quantitative PCR (qPCR)

Harvested mycelia were homogenized in 1 mL peqGOLD TriFast DNA/RNA/protein purification system (PEQLAB Biotechnologie, Erlangen, Germany) using a FastPrep FP120 BIO101 ThermoSavant cell disrupter (Qbiogene, Carlsbad, US). RNA was isolated according to the manufacturer's instructions.

The synthesis of cDNA from mRNA was achieved with the RevertAid™H Minus First Strand cDNA Synthesis Kit (Fermentas, St. Leon-Rot, Germany).

All PCRs for checking transcription were performed in an iCycler iQ, Real-Time Detection System (Bio-Rad, Hercules, US). The reactions were performed in a 25 μl volume containing 1 × buffer (Promega), 2.5 mM MgCl_2_, 0.1 μM forward primer, 0.1 μM reverse primer, 0.25 U Taq-polymerase (Go-taq, Promega), and a mixture of glucose and xylose-derived cDNAs (10-fold diluted) as template. The primer sequences (transkr2488f/r, transkr3151f/r, transkr3151f/r, transkr7236f/r) are given in Table 1. The PCR run included a blank (sterile, bi-distilled water instead of sample). The following PCR protocol was used: 3 min initial denaturation at 95°C, followed by 30 cycles of 15 s at 95°C, 15 s at 59°C, and 20 s at 72°C.

All qPCRs were performed in a Mastercycler^® ^ep realplex^2 ^(Eppendorf, Hamburg, Germany). The software realplex 2.2 was used to compile PCR protocols and define plate set-ups. All reactions were performed in triplicate. To analyze *2488prp *transcription, a SYBR Green assay with an actin reference was performed using 1 × iQ SYBR Green Supermix (Bio-Rad), 0.1 μM forward primer, 0.1 μM reverse primer, and cDNA as template. Primer pairs are given in Table 1. The following PCR program was used: 3 min initial denaturation at 95°C, followed by 45 cycles of 15 s at 95°C, 15 s at 59°C and 15 s at 72°C. The data are expressed relative to the transcription of the actin gene. The data in the figures are means of three independent experiments. Error bars indicate standard deviations. These amounts always refer to one reference sample within an experiment, which is marked in the figure with an asterisk.

### Isolation of DNA-binding proteins with streptavidin affinity chromatography

Biotinylated oligonucleotides (VBC, Vienna, Austria) (Bxyn2p250f/r, see Table 1) were annealed (95°C, 5 min followed by a slow cool to 35°C and then ice) and incubated with 200 μL μMACS Streptavidin Micro Beads (Miltenyi Biotec GmbH, Bergisch Gladbach, Germany) on ice for 15 min. Afterwards, 800 μL of binding mix (200 mM KCl, 200 ng/μL poly(dIdC), 200 ng/μL CKT067/CKT068, 8 mM Spermidine) and then 880 μL of cell-free extract protein were added and incubated on ice for 15 min first and then for 10 min. CKT067/CKT068 is a double-stranded DNA fragment used to titrate proteins that nonspecifically interact with DNA. This fragment and poly(dIdC) is used in a ten-fold excess of the specific probe [41]. Cell-free extracts were prepared as described previously [41]. Magnetic separation was performed according to the manufacturer's instructions after equilibration and subsequent saturation with CKT067/068 was achieved. Elution was performed with 150 μL of cold (room temperature) SDS loading buffer followed by 150 μL of hot (60 to 70°C) SDS loading buffer. The eluate was placed on ice immediately.

### Nano-HPLC-separation and identification of target proteins

Prior to MS analysis, reversed phase (RP) nano-HPLC separation of the eluted peptide mixtures from an in gel digest was performed. An UltiMate™HPLC System equipped with a FAMOS autosampler, Switchos and UV detector (all Dionex, Sunnyvale, CA, US) was used. After concentration and desalting on a trapping column (Acclaim PepMap100 C18, 5 μm, 100 Å, 300 μm i.d. × 5 mm, Dionex), peptides were separated on a PepMap 100 (C18) nanocolumn (Acclaim PepMap100 C18, 3 μm, 100 Å 75 μm i.d. × 15 cm, Dionex) at a flow-rate of 275 nL/min. Peptides were eluted with a linear gradient from 0% to 50% B in 30 min formed by mixing the two solvents A (5% ACN, 0.1% FA) and B (80% ACN, 0.08% FA), followed by a high organic wash (4 min at 90% B). The quality of separation was monitored by UV absorption at 214 nm.

The outlet of the nano-HPLC system was directly coupled to a Thermo™LTQ linear ion trap mass spectrometer (Thermo Electron Corp., Waltham, MA, US). Mass spectra were acquired in positive ionization mode. The applied method consisted of seven scans; the first was used to determine the precursor ions that were investigated in the following tandem mass scans by CAD (collisionally activated dissociation) fragmentation.

### Database searching

Tandem mass spectra were extracted by extract-msn (Thermo). Charge state deconvolution and deisotoping were not performed. All MS/MS samples were analyzed using Mascot (Matrix Science, London, UK; version 2.2.04). Mascot was set up to search the treeseiV2_FrozenGeneCatalog20081022.proteins.fasta.gz database (unknown version, 9143 entries), using predictions for a trypsin digest. Mascot was searched with a fragment ion mass tolerance of 0.60 Da and a parent ion tolerance of 1.5 Da. The iodoacetamide derivative of cysteine was specified in Mascot as a fixed modification. S-carbamoylmethylcysteine cyclization of the N-terminus and methionine oxidation were specified in Mascot as variable modifications.

### Criteria for protein identification

Scaffold (version Scaffold-01_07_00, Proteome Software Inc., Portland, OR) was used to validate MS/MS based peptide and protein identifications. Peptide identifications were accepted if they could be established at greater than 95.0% probability as specified by the Peptide Prophet algorithm [42]. Protein identifications were accepted if they could be established at greater than 99.0% probability and contained at least three identified peptides. Protein probabilities were assigned by the Protein Prophet algorithm [43]. Proteins that contained similar peptides and could not be differentiated based on MS/MS analysis alone were grouped to satisfy the principles of parsimony.

### Vector construction

Vectors for expressing the DNA-binding domains (DBD) as GST fusion proteins were constructed as follows. PCR was used to amplify the DBD coding regions using 25 μl reaction-mixtures containing 1 × buffer with MgCl_2 _(Fermentas), 0.1 μM forward primer, 0.1 μM reverse primer, 0.5 U High Fidelity DNA Polymerase (Fermentas), and genomic DNA as the template. Primer sequences (Exp2488F/R, Exp3151F/R, and Exp7236F/R) are given in Table 1. The following PCR protocol was followed: 3 min initial denaturation at 95°C, followed by 30 cycles of 15 s at 95°C, 15 s at 59°C, and 15 s at 72°C. Derived amplicons (165 bp, 219 bp, and 258 bp) were inserted into plasmid pGEX-4T-2 (Amersham) via the restriction enzymes BamHI and XhoI to obtain p2488DBD, p3151DBD, and p7236DBD, respectively.

The plasmid for expressing the 2488prp full length protein as an *in vitro *translation product was constructed as follows. PCR was used to amplify full length cDNA as described above, but in this case, the template was a mixture of cDNAs obtained under repressing conditions (glucose). The primer sequences (fltn2488f/r) are given in Table 1. The PCR protocol was as follows: 3 min initial denaturation at 95°C, followed by 30 cycles of 15 s at 95°C, 15 s at 59°C, and 2 min at 72°C. The derived amplicon (1,479 bp) was inserted into plasmid pTNT (Promega) via the restriction enzyme KpnI and NotI to obtain pMPF2488.

### Purification of the DBDs of 2488prp, 3151prp, and 7236prp as GST fusion proteins

All heterologously expressed proteins used in this study were produced as GST fusions with the pGEX system (Amersham), following the manufacturer's guidelines and using the plasmids p2488DBD, p3151DBD, and p7236DBD as templates. Expression products purified via glutathione-sepharose-columns (Amersham) were verified by SDS-PAGE (using the Mini-PROTEAN System, Bio-Rad) followed by SYPRO Ruby (Bio-Rad) staining. Thrombin cleavage was performed for 1 h at 37°C with 1 U of thrombin (Amersham), 150 mM NaCl, 2.5 mM CaCl_2_, and 25 mM Tris/HCl (pH 8) plus 20% Glycerin to remove the GST moiety.

## Authors' contributions

ARMA carried out the EMSAs, streptavidin affinity chromatography assays, transcript analyses, and drafted the manuscript. KGH carried out the Nano-HPLC/tandem MS-coupled detection and participated in the database search. MJPF carried out the vector construction and expression of GST fusion proteins. KM participated in the database search and data interpretation. RLM participated in the conception of the study and revised the manuscript. All authors read and approved the final manuscript

## Supplementary Material

Additional file 1 **List of *H. jecorina *proteins identified by tandem MS analysis**. The corresponding proteomic data are available at the publically available database https://proteomecommons.org/. Click here for file

Additional file 2 **Multiple sequence alignment of the *H. jecorina *2488prp with hypothetical proteins of other fungi, namely *Nectria haematococca *(GeneID: 9678588), *Gibberella zeae *(GeneID: 2791570), *Neurospora crassa *(GeneID: 3874038), *Podospora anserina *(GeneID: 6189947) *Chaetomium globosum *(GeneID: 4395593), *Magnaporthe grisea *(GeneID: 2675104)**. Click here for file
