# Supplementary material for: From an electrophoretic mobility shift assay to isolated transcription factors: a fast genomic-proteomic approach
Source: BMC Genomics. 2010 Nov 18;11:644. doi: 10.1186/1471-2164-11-644 (PMC3012607; doi:10.1186/1471-2164-11-644)
Supplement: Additional file 1 — List of H. jecorina proteins identified by tandem MS analysis. The corresponding proteomic data are available at the publically available database https://proteomecommons.org/. [file 1471-2164-11-644-S1.PDF]

Probability Legen..
- over 95%
- 80% to 94%
- 50% to 79%
- 20% to 49%
- 0% to 19%

#	Visible?	Protein Starred?	Bio View: Identified Proteins (81)	Accession Number	Molecular Weight	WT_sol_dig
1	☑	☆	**jgi|Trire2|44504|estExt_Genewise1.C_11839**	jgi|Trire2|44504|estExt_Genewise1.C_11839	42 kDa	20
2	☑	☆	**jgi|Trire2|21957|estExt_fgenesh1_pm.C_50191**	jgi|Trire2|21957|estExt_fgenesh1_pm.C_50191	131 kDa	19
3	☑	☆	**jgi|Trire2|122572|estExt_fgenesh5_pg.C_130159**	jgi|Trire2|122572|estExt_fgenesh5_pg.C_130159	67 kDa	19
4	☑	☆	**jgi|Trire2|81110|estExt_GeneWisePlus.C_220208**	jgi|Trire2|81110|estExt_GeneWisePlus.C_220208	256 kDa	19
5	☑	☆	**jgi|Trire2|55362|e_gw1.2.175.1**	jgi|Trire2|55362|e_gw1.2.175.1	73 kDa	18
6	☑	☆	**jgi|Trire2|46958|estExt_Genewise1.C_60370**	jgi|Trire2|46958|estExt_Genewise1.C_60370	50 kDa	17
7	☑	☆	**jgi|Trire2|123114|estExt_fgenesh5_pg.C_180087**	jgi|Trire2|123114|estExt_fgenesh5_pg.C_180087	79 kDa	17
8	☑	☆	**jgi|Trire2|22994|estExt_fgenesh1_pm.C_200019**	jgi|Trire2|22994|estExt_fgenesh1_pm.C_200019	90 kDa	12
9	☑	☆	**jgi|Trire2|62100|e_gw1.9.287.1**	jgi|Trire2|62100|e_gw1.9.287.1	23 kDa	12
10	☑	☆	**jgi|Trire2|123071|estExt_fgenesh5_pg.C_170219**	jgi|Trire2|123071|estExt_fgenesh5_pg.C_170219	55 kDa	12
11	☑	☆	**jgi|Trire2|55060|e_gw1.2.720.1**	jgi|Trire2|55060|e_gw1.2.720.1	103 kDa	9
12	☑	☆	**jgi|Trire2|46619|estExt_Genewise1.C_50587**	jgi|Trire2|46619|estExt_Genewise1.C_50587	45 kDa	8
13	☑	☆	**jgi|Trire2|122886|estExt_fgenesh5_pg.C_160102**	jgi|Trire2|122886|estExt_fgenesh5_pg.C_160102	50 kDa	8
14	☑	☆	**jgi|Trire2|79405|estExt_GeneWisePlus.C_130412**	jgi|Trire2|79405|estExt_GeneWisePlus.C_130412	98 kDa	8
15	☑	☆	**jgi|Trire2|103756|fgenesh5_pg.C_scaffold_2000344**	jgi|Trire2|103756|fgenesh5_pg.C_scaffold_2000344	118 kDa	8
16	☑	☆	**jgi|Trire2|78423|estExt_GeneWisePlus.C_100460**	jgi|Trire2|78423|estExt_GeneWisePlus.C_100460	122 kDa	8
17	☑	☆	**jgi|Trire2|120053|estExt_fgenesh5_pg.C_20133**	jgi|Trire2|120053|estExt_fgenesh5_pg.C_20133	73 kDa	7
18	☑	☆	**jgi|Trire2|119731|estExt_fgenesh5_pg.C_10398**	jgi|Trire2|119731|estExt_fgenesh5_pg.C_10398	61 kDa	7
19	☑	☆	**jgi|Trire2|23206|estExt_fgenesh1_pm.C_250029**	jgi|Trire2|23206|estExt_fgenesh1_pm.C_250029	51 kDa	7
20	☑	☆	**jgi|Trire2|73934|estExt_GeneWisePlus.C_10789**	jgi|Trire2|73934|estExt_GeneWisePlus.C_10789	34 kDa	7
21	☑	☆	**jgi|Trire2|21673|estExt_fgenesh1_pm.C_30234**	jgi|Trire2|21673|estExt_fgenesh1_pm.C_30234	59 kDa	7
22	☑	☆	**jgi|Trire2|123705|estExt_fgenesh5_pg.C_250090**	jgi|Trire2|123705|estExt_fgenesh5_pg.C_250090	68 kDa	7
23	☑	☆	**jgi|Trire2|121801|estExt_fgenesh5_pg.C_90164**	jgi|Trire2|121801|estExt_fgenesh5_pg.C_90164	66 kDa	7
24	☑	☆	**jgi|Trire2|21564|estExt_fgenesh1_pm.C_30019**	jgi|Trire2|21564|estExt_fgenesh1_pm.C_30019	85 kDa	7
25	☑	☆	**jgi|Trire2|123946|estExt_fgenesh5_pg.C_290010**	jgi|Trire2|123946|estExt_fgenesh5_pg.C_290010	32 kDa	6
26	☑	☆	**jgi|Trire2|76215|estExt_GeneWisePlus.C_50035**	jgi|Trire2|76215|estExt_GeneWisePlus.C_50035	49 kDa	6
27	☑	☆	**jgi|Trire2|120235|estExt_fgenesh5_pg.C_20496**	jgi|Trire2|120235|estExt_fgenesh5_pg.C_20496	93 kDa	6
28	☑	☆	**jgi|Trire2|80881|estExt_GeneWisePlus.C_210110**	jgi|Trire2|80881|estExt_GeneWisePlus.C_210110	48 kDa	6
29	☑	☆	**jgi|Trire2|49213|estExt_Genewise1.C_120252**	jgi|Trire2|49213|estExt_Genewise1.C_120252	30 kDa	6
30	☑	☆	**jgi|Trire2|76617|estExt_GeneWisePlus.C_50831**	jgi|Trire2|76617|estExt_GeneWisePlus.C_50831	62 kDa	6
31	☑	☆	**jgi|Trire2|77142|estExt_GeneWisePlus.C_70398**	jgi|Trire2|77142|estExt_GeneWisePlus.C_70398	145 kDa	6
32	☑	☆	**jgi|Trire2|74123|estExt_GeneWisePlus.C_11137**	jgi|Trire2|74123|estExt_GeneWisePlus.C_11137	74 kDa	6
33	☑	☆	**jgi|Trire2|120789|estExt_fgenesh5_pg.C_40423**	jgi|Trire2|120789|estExt_fgenesh5_pg.C_40423	50 kDa	5
34	☑	☆	**jgi|Trire2|123244|estExt_fgenesh5_pg.C_190118**	jgi|Trire2|123244|estExt_fgenesh5_pg.C_190118	58 kDa	5
35	☑	☆	**jgi|Trire2|77587|estExt_GeneWisePlus.C_80489**	jgi|Trire2|77587|estExt_GeneWisePlus.C_80489	47 kDa	5
36	☑	☆	**jgi|Trire2|105623|fgenesh5_pg.C_scaffold_5000408**	jgi|Trire2|105623|fgenesh5_pg.C_scaffold_5000408	33 kDa	5
37	☑	☆	**jgi|Trire2|4308|fgenesh1_pm.C_scaffold_11000125**	jgi|Trire2|4308|fgenesh1_pm.C_scaffold_11000125	99 kDa	5
38	☑	☆	**jgi|Trire2|80326|estExt_GeneWisePlus.C_180038**	jgi|Trire2|80326|estExt_GeneWisePlus.C_180038	51 kDa	5
39	☑	☆	**jgi|Trire2|122879|estExt_fgenesh5_pg.C_160091**	jgi|Trire2|122879|estExt_fgenesh5_pg.C_160091	54 kDa	5
40	☑	☆	**jgi|Trire2|44419|estExt_Genewise1.C_11713**	jgi|Trire2|44419|estExt_Genewise1.C_11713	58 kDa	5
41	☑	☆	**jgi|Trire2|46702|estExt_Genewise1.C_50722**	jgi|Trire2|46702|estExt_Genewise1.C_50722	49 kDa	5
42	☑	☆	**jgi|Trire2|80142|estExt_GeneWisePlus.C_170155**	jgi|Trire2|80142|estExt_GeneWisePlus.C_170155	103 kDa	5
43	☑	☆	**jgi|Trire2|120418|estExt_fgenesh5_pg.C_30308**	jgi|Trire2|120418|estExt_fgenesh5_pg.C_30308	138 kDa	4
44	☑	☆	**jgi|Trire2|52553|estExt_Genewise1.C_340053**	jgi|Trire2|52553|estExt_Genewise1.C_340053	205 kDa	4
45	☑	☆	**jgi|Trire2|120378|estExt_fgenesh5_pg.C_30213**	jgi|Trire2|120378|estExt_fgenesh5_pg.C_30213	40 kDa	4
46	☑	☆	**jgi|Trire2|108909|fgenesh5_pg.C_scaffold_13000255**	jgi|Trire2|108909|fgenesh5_pg.C_scaffold_13000255	35 kDa	4
47	☑	☆	**jgi|Trire2|80806|estExt_GeneWisePlus.C_200299**	jgi|Trire2|80806|estExt_GeneWisePlus.C_200299	68 kDa	4
48	☑	☆	**jgi|Trire2|74983|estExt_GeneWisePlus.C_20875**	jgi|Trire2|74983|estExt_GeneWisePlus.C_20875	41 kDa	4
49	☑	☆	**jgi|Trire2|79686|estExt_GeneWisePlus.C_140453**	jgi|Trire2|79686|estExt_GeneWisePlus.C_140453	81 kDa	4
50	☑	☆	**jgi|Trire2|50542|estExt_Genewise1.C_180007**	jgi|Trire2|50542|estExt_Genewise1.C_180007	68 kDa	4
51	☑	☆	**jgi|Trire2|80167|estExt_GeneWisePlus.C_170192**	jgi|Trire2|80167|estExt_GeneWisePlus.C_170192 (+1)	11 kDa	4
52	☑	☆	**jgi|Trire2|120064|estExt_fgenesh5_pg.C_20153**	jgi|Trire2|120064|estExt_fgenesh5_pg.C_20153	71 kDa	4
53	☑	☆	**jgi|Trire2|105190|fgenesh5_pg.C_scaffold_4000568**	jgi|Trire2|105190|fgenesh5_pg.C_scaffold_4000568	147 kDa	4
54	☑	☆	**jgi|Trire2|45604|estExt_Genewise1.C_30796**	jgi|Trire2|45604|estExt_Genewise1.C_30796	21 kDa	4
55	☑	☆	**jgi|Trire2|123471|estExt_fgenesh5_pg.C_220060**	jgi|Trire2|123471|estExt_fgenesh5_pg.C_220060	46 kDa	4
56	☑	☆	**jgi|Trire2|74774|estExt_GeneWisePlus.C_20435**	jgi|Trire2|74774|estExt_GeneWisePlus.C_20435	32 kDa	4
57	☑	☆	**jgi|Trire2|121826|estExt_fgenesh5_pg.C_90199**	jgi|Trire2|121826|estExt_fgenesh5_pg.C_90199	53 kDa	3
58	☑	☆	**jgi|Trire2|21742|estExt_fgenesh1_pm.C_40092**	jgi|Trire2|21742|estExt_fgenesh1_pm.C_40092	50 kDa	3
59	☑	☆	**jgi|Trire2|79419|estExt_GeneWisePlus.C_130435**	jgi|Trire2|79419|estExt_GeneWisePlus.C_130435	39 kDa	3

Probability Legend:
- over 95%
- 80% to 94%
- 50% to 79%
- 20% to 49%
- 0% to 19%

#	Visible?	Protein Starred?	Bio View: Identified Proteins (81)	Accession Number	Molecular Weight	WT_sol_dig
60	☑	☆	**jgi|Trire2|78401|estExt_GeneWisePlus.C_100413**	jgi|Trire2|78401|estExt_GeneWisePlus.C_100413	118 kDa	3
61	☑	☆	**jgi|Trire2|121901|estExt_fgenesh5_pg.C_90330**	jgi|Trire2|121901|estExt_fgenesh5_pg.C_90330	98 kDa	3
62	☑	☆	**jgi|Trire2|71363|kg2.C_scaffold_1000148**	jgi|Trire2|71363|kg2.C_scaffold_1000148	116 kDa	3
63	☑	☆	**jgi|Trire2|66436|e_gw1.18.322.1**	jgi|Trire2|66436|e_gw1.18.322.1	34 kDa	3
64	☑	☆	**jgi|Trire2|80920|estExt_GeneWisePlus.C_210182**	jgi|Trire2|80920|estExt_GeneWisePlus.C_210182	38 kDa	3
65	☑	☆	**jgi|Trire2|21557|estExt_fgenesh1_pm.C_20270**	jgi|Trire2|21557|estExt_fgenesh1_pm.C_20270	72 kDa	3
66	☑	☆	**jgi|Trire2|73774|estExt_GeneWisePlus.C_10526**	jgi|Trire2|73774|estExt_GeneWisePlus.C_10526	74 kDa	3
67	☑	☆	**jgi|Trire2|102870|fgenesh5_pg.C_scaffold_1000503**	jgi|Trire2|102870|fgenesh5_pg.C_scaffold_1000503	152 kDa	3
68	☑	☆	**jgi|Trire2|54454|e_gw1.1.564.1**	jgi|Trire2|54454|e_gw1.1.564.1	48 kDa	3
69	☑	☆	**jgi|Trire2|123562|estExt_fgenesh5_pg.C_230105**	jgi|Trire2|123562|estExt_fgenesh5_pg.C_230105	52 kDa	3
70	☑	☆	**jgi|Trire2|64345|e_gw1.13.342.1**	jgi|Trire2|64345|e_gw1.13.342.1	63 kDa	3
71	☑	☆	**jgi|Trire2|35183|gw1.1.1237.1**	jgi|Trire2|35183|gw1.1.1237.1	100 kDa	3
72	☑	☆	**jgi|Trire2|73733|estExt_GeneWisePlus.C_10446**	jgi|Trire2|73733|estExt_GeneWisePlus.C_10446	59 kDa	3
73	☑	☆	**jgi|Trire2|121028|estExt_fgenesh5_pg.C_50371**	jgi|Trire2|121028|estExt_fgenesh5_pg.C_50371	30 kDa	3
74	☑	☆	**jgi|Trire2|61470|e_gw1.9.296.1**	jgi|Trire2|61470|e_gw1.9.296.1	22 kDa	3
75	☑	☆	**jgi|Trire2|23363|estExt_fgenesh1_pm.C_300033**	jgi|Trire2|23363|estExt_fgenesh1_pm.C_300033	30 kDa	3
76	☑	☆	**jgi|Trire2|73678|estExt_GeneWisePlus.C_10338**	jgi|Trire2|73678|estExt_GeneWisePlus.C_10338	62 kDa	3
77	☑	☆	**jgi|Trire2|64193|e_gw1.13.303.1**	jgi|Trire2|64193|e_gw1.13.303.1	88 kDa	3
78	☑	☆	**jgi|Trire2|78817|estExt_GeneWisePlus.C_110490**	jgi|Trire2|78817|estExt_GeneWisePlus.C_110490	44 kDa	3
79	☑	☆	**jgi|Trire2|123155|estExt_fgenesh5_pg.C_180159**	jgi|Trire2|123155|estExt_fgenesh5_pg.C_180159	26 kDa	3
80	☑	☆	**jgi|Trire2|21425|estExt_fgenesh1_pm.C_20025**	jgi|Trire2|21425|estExt_fgenesh1_pm.C_20025	49 kDa	3
81	☑	☆	**jgi|Trire2|46708|estExt_Genewise1.C_50728**	jgi|Trire2|46708|estExt_Genewise1.C_50728	137 kDa	3
