# Supplementary material for: From an electrophoretic mobility shift assay to isolated transcription factors: a fast genomic-proteomic approach
Source: BMC Genomics. 2010 Nov 18;11:644. doi: 10.1186/1471-2164-11-644 (PMC3012607; doi:10.1186/1471-2164-11-644)
Supplement: Additional file 2 — Multiple sequence alignment of the H. jecorina 2488prp with hypothetical proteins of other fungi, namely Nectria haematococca (GeneID: 9678588), Gibberella zeae (GeneID: 2791570), Neurospora crassa (GeneID: 3874038), Podospora anserina (GeneID: 6189947) Chaetomium globosum (GeneID: 4395593), Magnaporthe grisea (GeneID: 2675104). [file 1471-2164-11-644-S2.PDF]

Results colour-coded for amino acid conservation

The current colourscheme of the alignment is for **amino acid conservation**.

The conservation scoring is performed by PRALINE. The scoring scheme works from 0 for the least conserved alignment position, up to 10 for the most conserved alignment position. The colour assignments are:

Unconserved 0 1 2 3 4 5 6 7 8 9 10 **Conserved**

```
                 .........10 .........20 .........30 .........40 .........50
Hypocrea_jecori  MAQALDISSS SGPGPLVALP E-RTLGPPAS AITPPSTVQ- --------RS
Nectria_haemato  MDPNL-L--- S----FFSVP NNQTLNPVDQ NAESSTGAIS QQQQQPQEPS
Gibberella_zeae  MDPNI-L--- N----FFAVP NNQTLNNVDQ SNQSSQQQHE --------PS
Neurospora_cras  MDPNT-LRHL E----YFSVP NHQTVNPVDQ SYNLSGSTST QS------PT
Magnaporthe_ory  MDPNL-LRHM E----YFTVP NAQTRNAAER AANLTRSSGT GQ------PY
Podospora_anser  MDSDP-LRHL E----FFSMP NNQTVNPVDQ PFMTSTSAQA --------PP
Chaetomium_glob  ---------- ---------- ---------- ---------- ----------
Consistency      7544306111 4000045457 5267453444 3222423322 0000000053

                 .........60 .........70 .........80 .........90 .........100
Hypocrea_jecori  A-WTSSPRAP AMDMFNFTTA ASASAVASY- -APAGGSNSN SNSNSGGNSG
Nectria_haemato  P-WRP----L EGDMVDRTSG PQGGPVSSY- -DHQSSAPSD ITFTQAPMPS
Gibberella_zeae  P-WGP----A DLESGMGASR NQGASSFGF- -QPTSTSDI- -SFTQPPMQS
Neurospora_cras  L-W-----GT APSLEANHSG MNSTATTTMS LDSLSQTSPA TAFTGMSSHP
Magnaporthe_ory  PLWNPSTLGA DIDFSFNPEG LGGMGFGAFE -QPQDQNNSS TAFEPPQRQR
Podospora_anser  I-WNTTGMGL DGGISMSPST APTPPSLDS- -IAQPSSLTG FNSIPPGISS
Chaetomium_glob  ---------- ---------- ---------- ---MSHDVSS RSNLPAQGIQ
Consistency      2072210023 3133222253 2232332330 0234545353 2544443334

                 .........110.........120.........130.........140.........150
Hypocrea_jecori  GLGDMNFSQM PQYSGRNPLQ SMLSQTKEGP DAKRIKVESP H-SALDSIDS
Nectria_haemato  PYTQH-KTVL PTRG--HPRT HPRKRD--AH DRKRTKVDTE S--ALESLDY
Gibberella_zeae  TYTGPNNSIL PNRGHPRNNP QQRRRDGHAH DRKRTKVDTD S--ALESLDY
Neurospora_cras  SMTRRPSMYG NHTGMPS-SK SSRTRE--GH ERKRTRLDSE L-SPFDEPDY
Magnaporthe_ory  S----IYTQG PRTLVPTRTP KPPSRE--KP ERKRSKLSTD S-TPFDSVDY
Podospora_anser  ASQASTHSRQ PRPVLPARGV RRDAHE---- -RKRSRLSMD A-TPLDSVDY
Chaetomium_glob  SR-----SVL PARG----QR SRDGHE---- -RKRSRLSLD TNTPLDSVDY
Consistency      5221112633 7434132133 5334660022 38**687656 40367886*7

                 .........160.........170.........180.........190.........200
Hypocrea_jecori  WLQFDEDADK FGS------- ----FEIDFS KQYNTANQPR ASTNM-----
Nectria_haemato  WIQFDDDEAE RT-----GSY EIDFSKRYDM MNNTTTTR-P GFGSS-----
Gibberella_zeae  WIRFDDDEAE KA-----GSY EIDFSKRYDP MSNANTHYRP GYGSS-----
Neurospora_cras  WLQFDNDDNF DDLSEITEPP RQDHKGKGRA PQLSQQQEQQ QQQQQQRRRS
Magnaporthe_ory  WLQFDNEGTI PE--ETSAEA GATTQGEPSA ESKVQEPEPQ RSHK-----Q
Podospora_anser  WLDFDKDEGL AS-------- ---IPEGVEA SRREGELRPP VKRSS-----
Chaetomium_glob  WIDFDNDDSL AS-------- ---IPEAFEP RSTAKVKKNK APMR------
Consistency      *86**58543 2400000110 1112343244 3534442534 3335200000

                 .........210.........220.........230.........240.........250
Hypocrea_jecori  ---TPGLGSG LYANPTAPFR EEDFLDDSAF DHSLSEDDEM FDN-ININDQ
Nectria_haemato  --STTPGLGT GIYATAPPFK GNDYFDDNAL DNALSDDEEG -FDSMSLEEH
Gibberella_zeae  --SNTPGLGT GVYANSLPFQ GAEYFDDNAL DNALSDEEEE GIDSMSLEEH
Neurospora_cras  TAHSMATTAP THRRTAGTLK SEDYMEDSAL DNALSEDERA -MS-LNLAEQ
Magnaporthe_ory  KQQGEARAAS QQGSRTAQLK VEDSLDDSAL ENALSDDEQT -FASISLAEQ
Podospora_anser  --RNTAHAGG RLKHEEMMDD SALDNALSDD DDDDDGFSSI -----NLADH
Chaetomium_glob  -------RPT NLAPSISTAR SDEIVDDSAL DNALSDEDGF SS--VNLTDQ
Consistency      0023331354 3333443445 4554467775 8767866752 0221579476

                 .........260.........270.........280.........290.........300
Hypocrea_jecori  LSKLGTLPST EAQSSRSLFS TPSMGWEKPQ QGVQPSAVMG DEIPRRMTSD
Nectria_haemato  LSKIETMPPP EVPPREGLYS TP-LSWEKPE PGLRMEPNFG VGESPGGMST
Gibberella_zeae  LSKIETMPPP EVPPREGLYS TP-LSWEKPE PGIRMEPDFG MGSTQGAMNL
Neurospora_cras  LSKIDTAPPQ EVPPREGLYS TP-LSWERPQ PGLRMDSLIG LN-QQAL---
Magnaporthe_ory  LSKIDSTAPT EIPQRESLFS TP-LSWERPQ PGLRMDSVMS TQSRSTL---
Podospora_anser  MSMIESAPPP EVPPREGLYS TP-LSWERPQ PGLRMDSLIG LNNQALNEA-
Chaetomium_glob  LSQINTLPPL EVPPREGLYS TP-LSWERPQ PGIHMDSPTG PGLSQ-----
Consistency      9*79585884 *886887*8* **098**8*8 8*87776358 3424422110

                 .........310.........320.........330.........340.........350
Hypocrea_jecori  SW--DPIPQG TNYTLTPDER RRLLEIAMGP GRLASYVNPS RFNMG-FGST
Nectria_haemato  AGYGQTMPGV GNQVLSNDEQ RRLLAIAMNT GRTPASFMPP SGFGLGFGAG
Gibberella_zeae  GFGQAMSGMG NNQVLSNEEQ RRLLAIAMNT GRTSSSFMPA TGFGLGFGAG
Neurospora_cras  ---------- -----NEAEQ RRLIAIAMNP GPSMGGLGSN INLNF---GD
```

```
Magnaporthe_ory ---------- -----DQAEQ RRLIAIAMNR GTTSGGLGSS MADQY---SQ
Podospora_anser ---------- EQRRLIAIAM NPGSSMGGLG SNLNLNFTGL NPGMS----T
Chaetomium_glob ---------- -----KEAEQ RRLIAIAMNP RSTMGGLGLG AGFDF-----
Consistency     0000000000 0211244487 8776788753 6464446333 3333301122

                .........360.........370.........380.........390.........400
Hypocrea_jecori MQPSISQEFG FGFGAPKQGL SHMGMYQRNN SADTSASPLS QSRELKHKQP
Nectria_haemato LGSGLPPEFN ASIDSILGTP GEQSQKQTPT PADSTKAPSR NQDIKTEASS
Gibberella_zeae LGTGMSDFNS NIESLLGTPT GDRSQKQTPT PTNSSKAPSR NQDIKTENSS
Neurospora_cras MATGFNSGFG SSYASSSMGG GQMPRPVSPP RASTSSQSRP APARPPHKHG
Magnaporthe_ory FQTDFGSAMS DFGLGTAS-- -------MSQ LLNMPPPNTV PPKQPQPQRL
Podospora_anser PFTAGGSGSG SMGMGMGMGT GMGMGMGMGG GMGGPKPVSP PHSTTPHPKP
Chaetomium_glob GGMGAGMPMA FGAGFEIPSI SHDPRPVSPP HLSGTQGRPG PSSLPKKRGG
Consistency     3355344345 3323333332 5222322443 2554544343 4443444443

                .........410.........420.........430.........440.........450
Hypocrea_jecori KVETPPKTVP TKRPSTLSET SSIGKEKVKS ADRMAHNDVE RKYRTNLKDK
Nectria_haemato ETGTALSSSR PQLHRSNTDT TDKGKDKMKS GDRTAHNDIE RKYRTNLKDK
Gibberella_zeae ETGLSSTRPG LSRTSTAT-S DVKGKEKLKL GDRTAHNDIE RKYRTNLKDK
Neurospora_cras SISEKSSERM GSGDKHRKVE EKH------KI GDRTAHNDIE RKYRTNLKDK
Magnaporthe_ory DKGKEK---- ---------- -AG------PK GDRTAHNDIE RKYRTNLKDK
Podospora_anser GSLHQPKRQG SVSDSKAKEK VKA------AG GDRTAHNDIE RKYRTNLKDK
Chaetomium_glob VGEKGEKAKE KP-------- ---------KT GERAAHNDIE RKYRTNLKDK
Consistency     3333334321 2211221212 1221111063 88*6****9* **********

                .........460.........470.........480.........490.........500
Hypocrea_jecori ITELRNAVPA LQQVAE---- ----GEPDAA QGIAKVSKGT VLTKATEYIH
Nectria_haemato IAELRNAVPA LQSIPE---D GI-DEGEGNS QRAPKVSKGT VLTKATEYIQ
Gibberella_zeae IAELRDAVPA LHSIPE---D GDNNEAEGDS QRAPKVSKGT VLTKATEYIQ
Neurospora_cras IAELREAVPA LHTISE---A GGVEDDGSQN SRAPKVSKGT ILTKATEYIH
Magnaporthe_ory ISELRDAVPA LRTIPENEED GEDAGQPSRG PKVSKVLQGA VLTKATEYIH
Podospora_anser ISELRDAVPS LRTIPE---- NEEGEDPNQP SRAPKVSKGA VLTKATEYIH
Chaetomium_glob ISELRDAVPA LRTILE---- EGDEDGETQP SRAAKISKGT VFTKAIEYIH
Consistency     *7***6***8 *5694*0001 4223544444 5675*978*7 98***8***7

                .........510.........520.........530.........540.........550
Hypocrea_jecori QLEQRNKDIM TEHKQLMRRL QAFEALLNNS MR-VDIMPSH SMTLFDPRGF
Nectria_haemato YLERRNKAIM KEHQELARRL QAFEQLLSAS ARQPFLMPNH SRTLFDPRGF
Gibberella_zeae HLERRNRAIM KEHQELARRL QAFEQLLSAS ARQPFLMPNH SRTLFDPRGF
Neurospora_cras QLERRNKQIT MEHRELSRKL QAFEQLLNHT AATAYQMPAY SRTLFDPRGF
Magnaporthe_ory YLEKKNKMIV QQHQELSKRL QAFEQLLSAT ARPTYQMPNY SRTLFDPRAF
Podospora_anser WLERRNKQIV QEHRELSRRL QAFEQLLNAT ARPQYMMPTY SRTLFDPRGF
Chaetomium_glob FLERQNKQIT QEHRNLSRRL QAFEQLLTVT AQQTYGMPAY TSALFDPRGF
Consistency     4**77*94*5 59*67*699* ****8**647 873463**57 868*****8*

                .
Hypocrea_jecori C
Nectria_haemato C
Gibberella_zeae C
Neurospora_cras C
Magnaporthe_ory C
Podospora_anser C
Chaetomium_glob C
Consistency     *
```
